# Editorial: Mesechymal-Like Stem Cells in Osteoarthritis and Inflammation: The Priming Role of the Environment

**DOI:** 10.3389/fcell.2022.889210

**Published:** 2022-03-25

**Authors:** Elena Stocco, Christopher Z. Mosher, Helen H. Lu, Raffaele De Caro

**Affiliations:** ^1^ Section of Human Anatomy, Department of Neuroscience, University of Padova, Padova, Italy; ^2^ Biomaterials and Interface Tissue Engineering Laboratory, Department of Biomedical Engineering, Columbia University, New York, NY, United States

**Keywords:** osteoarthiritis, inflammation, aging, tissue engineering, mesenchymal stem cells, plasticity, diseased tissue secretome

Osteoarthritis (OA) is a complex and multifaceted disease characterized by low-grade inflammation, progressive cartilage damage, and bone remodelling. Its symptomatic hallmarks are pain and stiffness, loss of mobility, and subsequent functional disability (Syx et al., 2018). Aging, injury, and obesity represent the main predisposing factors to OA, each of which may trigger metabolic OA phenotypes such as the production of pro-inflammatory cytokines, adipokines, and reactive oxygen species (Batushansky et al., 2021; Belluzzi et al., 2019). Due to OA’s multifactorial aetiology and the lack of a full understanding of its pathogenesis, control over OA symptoms remains largely inadequate with the only effective and resolutive treatment being surgical intervention, as total joint replacement (Syx et al., 2018).

Research efforts to find a solution for OA patients canonically focus on unravelling both the pathways leading to OA and the “actors” involved in its onset and progression, as well as deciphering the occurrence and the development of dynamic interactions (i.e., biological, biochemical) within the joint. It was previously reported that resident stem cells may be primed by the pathological, inflammatory microenvironment with a consequent incomplete protective activity over OA (Stocco et al., 2019). Thus, the goal of this special issue is to investigate the intrinsic features and plasticity of joint resident stem cells, focusing on the interplay between cells and the inflamed environment, specifically the diseased tissue secretome; concomitant conditions including age and/or obesity are also explored. Elucidating the role of stem cells in different settings is crucial to the development of future therapies for OA. For instance, cell-microenvironment cross-talk may guide researchers through the use of autologous OA joint cells and/or stem cells for tissue engineering strategies. Despite representing a tempting source for a vanguard and personalized medicine (Stocco et al., 2014, 2016), the cell environment-mediated fingerprint is often overlooked.

Within this topic, Liu et al. and Feng et al. have focused on the OA cartilaginous compartment and considered the possible cross-talk between OA-chondrocytes (OAC) and mesenchymal stromal cells (OA-MSCs) during aging to decipher their involvement in the inflammatory microenvironment. Cartilage is primarily afflicted in OA, the underlying dynamic cellular and molecular interactions still need to be understood to unravel the cascade of events leading to the typical anatomo-morphological and physio-pathological features of the diseased tissue. Liu et al. suggest that OA-MSCs are the senescent component of articular cartilage, claiming they are responsible for fibrosis and become a source of joint inflammation. Furthermore, aging, stress, or injury induces proliferation and dedifferentiation of OACs, resulting in the production of normal cartilage stromal cells (NCSCs) that are involved in tissue repair and wound healing. However, repeated activation of NCSC-like cell replication in aged cartilage triggers replicative senescence, resulting in an abundance of OA-MSCs. The data demonstrates that these OA-MSCs are active in the production of pro-inflammatory cytokines and chemokines including IL 1β, IL 6, IL 8, and CXCL1, 5, and 6—all senescence-associated secretory phenotypes (SASPs) whose main targets are chondrocytes that, in turn, activate towards catabolism and apoptosis mechanisms. Together with SASPs, OA-MSCs (but not OA chondrocytes) also express greater levels of the senescence marker p16INK4a, further demonstrating their inclination toward cells death. In parallel, Feng et al. elucidated the contributory roles of Sonic Hedgehog (SHH) and Indian Hedgehog (IHH) gene expression in OA cartilage: study results highlight the driving behaviour of SHH gene expression (over IHH), where SHH expression is initiated in human NCSCs and increases in senescent OA-MSCs with aging. In MSCs, SHH activates proliferation and chondrogenesis, but also inflammation and replicative senescence (induced by SASPs) that cause tissue degeneration *via* OAC catabolism and apoptosis. Corroborating this hypothesis, SHH knocked-down OA-MSCs do not show catabolic or senescence marker expression, and their secretome does not influence OAC apoptosis; thus, SHH is a potential molecular target for OA therapy.

In tandem with age, obesity has also long been suggested as a possible risk factor for severe knee OA. However, the correlation between this metabolic condition and detrimental structural changes in knee cartilage is uncertain (Go et al., 2021). MSCs exert a fundamental role over a tissue’s homeostasis by sensing and reacting to environmental cues; thus, investigating and interpreting their behaviour in a High Fat Diet (HFD) condition unveil the physio-pathological mechanisms behind OA. The HFD typically induces elevated levels of pro-inflammatory cytokines; for instance, Bi et al. suggested a boost in serum CXCL2 levels caused by the HFD, thus determining an impairment in bone marrow MSCs behaviour characterized by a decrease in their adipogenic potential (mediated by Rac1 signalling), as well as increased migration and senescence mediated by enhanced production of ELMO1 and reactive oxygen species, respectively. Describing the existing correlations between HFD-induced inflammatory microenvironmental alterations and bone marrow MSCs response aids in outlining the critical features of OA pathophysiology and points toward potential OA treatment *via* immunomodulation.

In addition to pain, chronic low-grade inflammation is a distinctive feature of OA, predisposing joints to dysfunction and structural OA progression. However, recent evidence establishes inflammation as a hallmark of tissue healing, demonstrating its positive influence over the regulatory effects controlling tissue regeneration and repair (Li et al., 2021). Considering this, Bohaud et al. highlighted the complex, ambivalent role of macrophages in both OA development and tissue regeneration, as well as their dialogue with MSCs throughout these processes. The mechanisms these cells are involved in, including activation of their phenotypic switches to an OA-like state, are elucidated in an effort to define an effective cell-based therapy for tissue regeneration. Disclosure of the fundamental events triggering OA pathogenesis is required to develop an efficient target therapy with vanguard treatment options projected towards tissue regeneration and cell-based therapies. “OA is a whole joint disease” is an axiom to keep in mind to effectively approach OA. Thus, solely focusing on a specific molecular pathway could be misleading when looking for effective therapies which cannot ignore the multifaced nature and multi-tissue systems involved with OA initiation and development (Maqbool et al., 2021). The manuscripts within offer a glimpse into the complex nature of this disease, as well as potential treatment options on the horizon.
